# Phenotypic and transcriptional changes in peripheral blood mononuclear cells during alphavirus encephalitis in mice

**DOI:** 10.1128/mbio.00736-24

**Published:** 2024-05-02

**Authors:** Benjamin H. Nguyen, Maggie L. Bartlett, Elizabeth M. Troisi, Elise Stanley, Diane E. Griffin

**Affiliations:** 1 W. Harry Feinstone Department of Molecular Microbiology and Immunology, Johns Hopkins Bloomberg School of Public Health, Baltimore, Maryland, USA; Duke University School of Medicine, Durham, North Carolina, USA

**Keywords:** viral encephalitis, Sindbis virus, peripheral blood mononuclear cells, scRNAseq, inflammation

## Abstract

Sindbis virus (SINV) infection of mice provides a model system for studying the pathogenesis of alphaviruses that infect the central nervous system (CNS) to cause encephalomyelitis. While studies of human viral infections typically focus on accessible cells from the blood, this compartment is rarely evaluated in mice. To bridge this gap, single-cell RNA sequencing (scRNAseq) was combined with flow cytometry to characterize the transcriptional and phenotypic changes of peripheral blood mononuclear cells (PBMCs) from SINV-infected mice. Twenty-one clusters were identified by scRNAseq at 7 days after infection, with a unique cluster and overall increase in naive B cells for infected mice. Uninfected mice had fewer immature T cells and CCR9^+^ CD4 T cells and a unique immature T cell cluster. Gene expression was most altered in the Ki67^+^ CD8 T cell cluster, with chemotaxis and proliferation-related genes upregulated. Global analysis indicated metabolic changes in myeloid cells and increased expression of *Ccl5* by NK cells. Phenotypes of PBMCs and cells infiltrating the CNS were analyzed by flow cytometry over 14 days after infection. In PBMCs, CD8 and Th1 CD4 T cells increased in representation, while B cells showed a transient decrease at day 5 in total, Ly6a^+^, and naive cells, and an increase in activated B cells. In the brain, CD8 T cells increased for the first 7 days, while Th1 CD4 T cells and naive and Ly6a^+^ B cells continued to accumulate for 14 days. Therefore, dynamic immune cell changes can be identified in the blood as well as the CNS during viral encephalomyelitis.

**IMPORTANCE:**

The outcome of viral encephalomyelitis is dependent on the host immune response, with clearance and resolution of infection mediated by the adaptive immune response. These processes are frequently studied in mouse models of infection, where infected tissues are examined to understand the mechanisms of clearance and recovery. However, studies of human infection typically focus on the analysis of cells from the blood, a compartment rarely examined in mice, rather than inaccessible tissue. To close this gap, we used single-cell RNA sequencing and flow cytometry to profile the transcriptomic and phenotypic changes of peripheral blood mononuclear cells (PBMCs) before and after central nervous system (CNS) infection in mice. Changes to T and B cell gene expression and cell composition occurred in PBMC and during entry into the CNS, with CCL5 being a differentially expressed chemokine. Therefore, dynamic changes occur in the blood as well as the CNS during the response of mice to virus infection, which will inform the analysis of human studies.

## INTRODUCTION

Mosquito-borne viruses, particularly alphaviruses and flaviviruses, are major causes of epidemic febrile, arthritic, and encephalitic diseases, and these viruses are of increasing concern to human populations due to the expansion of their mosquito vectors and introduction of viruses into new geographic regions (1–4). Encephalomyelitis is a particularly dangerous complication of alphavirus infection that can lead to acute fatal disease or recovery with long-term sequela, with clinical outcomes dependent on viral virulence and host immune responses (5, 6). As these viruses expand and adapt, they can become more neurovirulent, with the chikungunya virus being the most recent example from the alphavirus genus (7–9). There is currently no treatment for these infections.

Alphavirus-induced encephalomyelitis can be studied in a mouse model of infection with Sindbis virus (SINV), the prototypic alphavirus, which causes widespread infection of neurons. Acute disease consists of weight loss, hunched posture, and paralysis, with severity depending on both host genetics and the strain of SINV (10–14). Recovery from SINV infection of the central nervous system (CNS) involves resident neural cells and infiltrating leukocytes from the periphery (15). Due to the non-renewable nature of neurons, a successful immune response to viral infection of the CNS cannot depend on cytolytic mechanisms for control and is therefore specialized. Clearance of infectious virus and viral RNA from the CNS is accomplished by the adaptive immune response and involves recruitment of SINV-specific IFNγ-producing T cells and antibody-secreting cells (15).

The adaptive immune response to viral encephalomyelitis is initiated following antigen presentation in the draining cervical lymph nodes (CLNs) with subsequent recruitment of activated lymphocytes to the CNS. The appearance of lymphocytes in the CNS corresponds with the clearance of infectious virus and disease onset (16–18). Studies in mice typically focus on the tissue of interest, and in the context of encephalitic SINV infection, examination of brains and spinal cords has determined the mechanisms of non-cytolytic viral clearance. Peripheral blood mononuclear cells (PBMCs) are seldom examined in mice, despite the initiation of the adaptive immune response in the periphery. This contrasts with studies in humans, where the blood and serum samples are often used to test for or diagnose disease in a variety of organs including the CNS. However, present tests for viral encephalitis have poor sensitivity, specificity, and availability for the diagnosis of these diseases. For example, alphavirus infection in humans is diagnosed by testing serum for viral RNA or IgM or IgG antibodies, but awaiting results may delay intervention, resulting in higher mortality or more severe sequelae (19). Diagnosis may also involve testing cerebral spinal fluid, which is more difficult to collect than blood. Therefore, a better understanding of the changes in PBMCs during viral encephalomyelitis may provide insights into the induction of the adaptive immune response to CNS infection and reveal potential biomarkers for a more rapid and accurate diagnosis.

The CNS at steady state is generally void of immunologic activity and has low expression of major histocompatibility complex molecules, and the brain parenchyma contains no conventional lymphatic vessels or professional antigen-presenting cells (15). Therefore, the activation of lymphocytes in the periphery and their subsequent expansion, release from the CLNs, and trafficking into the CNS implies perturbations to PBMC composition associated with viral encephalitis. To investigate whether CNS-restricted, non-fatal SINV infection leads to transcriptional and immunologic changes in circulating PBMCs, single-cell RNA sequencing (scRNAseq) was performed on PBMCs from uninfected mice and infected mice 7 days after infection. Unique cell populations were identified, and changes to subtype composition were followed by flow cytometry over the course of infection. PBMC expression of inflammatory and proliferative genes and serum CCL5 levels were elevated following CNS infection. Therefore, CNS-restricted alphavirus infection is associated with transcriptomic, compositional, and immunological perturbations to PBMCs.

## RESULTS

### scRNAseq reveals transcriptomic and compositional changes in PBMCs following SINV infection of the CNS

**Fig 1:**
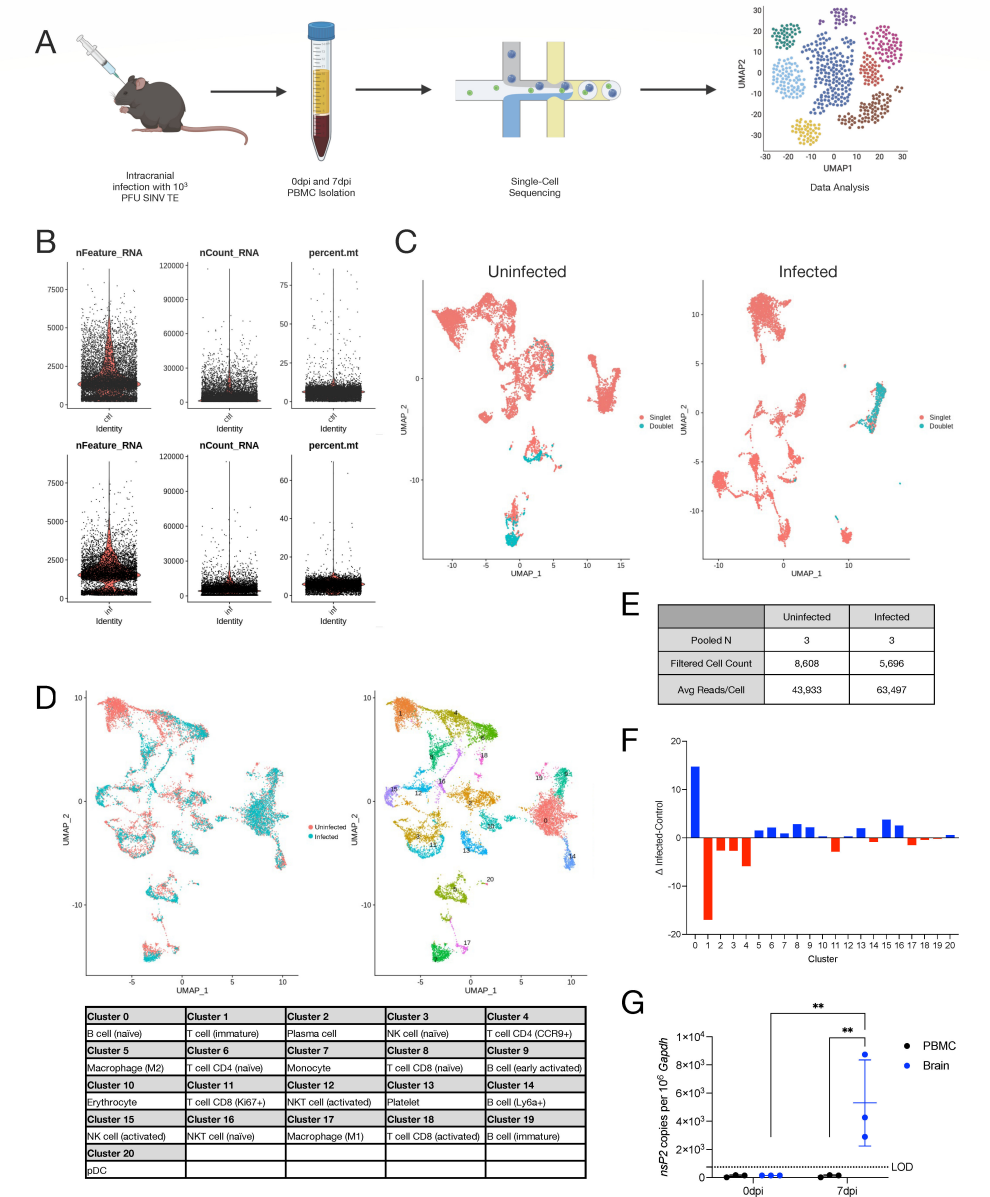
Single-cell RNA sequencing of PBMCs from SINV-infected mice. (**A**) Graphical abstract detailing experimental setup for single-cell RNA sequencing. (**B**) Quality control parameters examined for filtering were gene counts (left), read counts (center), and mitochondrial genes (right) for control (top) and infected (bottom) mice. (**C**) Doublets in the sequencing data that were filtered out visualized on a UMAP plot for control (left) and infected (right) mice. (**D**) Aggregate UMAP plot showing cell clusters present in circulating PBMCs striated by infected status (left) and cell type (right) with cell type identities corresponding to each cluster number (bottom). (**E**) Data table summarizing sample details and sequencing metrics. (**F**) Compositional changes for each cell type calculated as the difference between infected and control proportions for each cluster. (**G**) qRT-PCR for SINV *nsP2* expression in PBMCs and brains. Data are presented as the mean ± SD from two independent experiments with the cells pooled from four to seven mice. The limit of detection (LOD) is indicated with a dashed line. Samples with undetected transcripts were assigned a value of 150. No indicator, non-significant, *P < 0.05, **P < 0.01, ***P < 0.001, and ****P < 0.0001; Fisher’s LSD multiple comparisons test.

Young adult (4–6-week-old) C57BL6/J mice of both sexes were intracranially (i.c.) inoculated with 10^3^ PFU of the TE strain of SINV (12), and infection was allowed to progress for 7 days, the time of peak disease severity (20). Age- and sex-matched uninfected mice were used as controls. Heparinized blood was collected via cardiocentesis from three mice in each group and pooled. PBMCs were isolated for scRNAseq with an input of 11,200 cells from each sample (Fig. 1A). Data were preprocessed, cells were filtered based on mitochondrial gene representation and variances in unique gene counts, and doublets were removed (Fig. 1B and C), resulting in a total of 8,608 cells from uninfected mice and 5,696 cells from infected mice (Fig. 1E) used for downstream analyses via principal component analysis (PCA) and uniform manifold approximation and projection (UMAP) dimension reduction. Unsupervised clustering at a resolution determined by Clustree (21) analysis revealed 21 transcriptionally distinct clusters among circulating leukocytes in mice (Fig. 1D).

Each cell cluster was calculated as a proportion of the total cells in the sample. Subtraction of control from infected proportions identified changes to populations of circulating immune cells associated with SINV infection of the CNS (Fig. 1F). For example, cluster 0 (naive B cells) was about 15% higher, cluster 1 (immature T cells) was about 17% lower, and cluster 4 (*Ccr9*^+^ CD4 T cells) was about 6% lower in infected mice relative to uninfected mice. The remaining cluster proportions were basically unchanged with 0.1%–5% variation in either direction. Finally, to determine whether the SINV genome was present in circulating leukocytes, total RNA was isolated from brains and PBMCs from uninfected and infected mice at 7 dpi and analyzed by real-time quantitative reverse transcription PCR (RT-qPCR) for absolute copies of the SINV *nsP2* gene (Fig. 1G). Infected brains showed high levels of viral RNA copies, while PBMCs had no detectable viral transcripts at the same time point during infection (*P* < 0.01), demonstrating that intracranial inoculation with SINV results in an infection that is restricted to the CNS.

**Fig 2:**
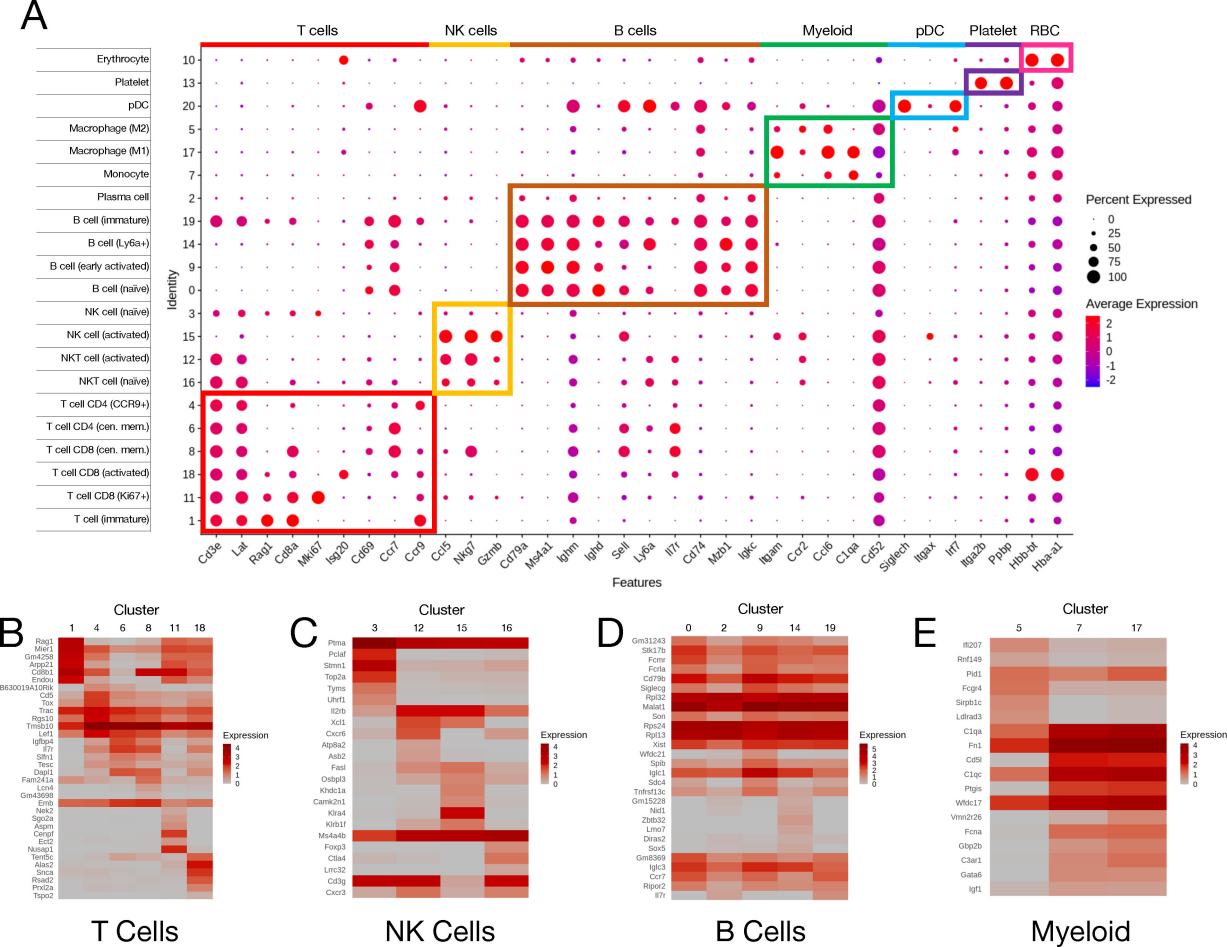
PBMC cluster annotation identifies seven major cell types. (**A**) DotPlot of markers used to identify super-cluster and subcluster identities. Dot size represents the frequency of cells expressing the gene in a cluster, while the color intensity represents the level of expression of that gene. Boxes highlight super-clusters and genes used to identify them and the subclusters within them. (**B–E**) Heatmaps of the top six genes used as markers for each cluster relative to the rest of the data set for T cells (**B**), NK cells (**C**), B cells (**D**), and myeloid cells (**E**).

Globally from both conditions, we identified six T cell (*Cd3e*^+^ and *Lat*^+^), four natural killer (NK) cell (*Nkg7*^+^ and *Gzmb*^+^), five B cell (*Cd79a*^+^ and *Ms4a1*^+^), and three myeloid cell (*Itgam*^+^) subpopulations based on automated cell-type query (22) with manual verification using known cellular markers (Fig. 2A). Within the T cells, we identified one immature cluster based on the expression of *Rag1*, three CD8 clusters consisting of central memory (*Ccr7*^+^, *Cd69*^+^, and *Sell*^+^) (23, 24), activated (*Isg20*^+^), and replicating (*Ki67*^+^), and three CD4 clusters consisting of central memory (*Ccr7*^+^, *Cd69*^+^, and *Sell*^+^), *Ccr9*^+^, and *Ccl5*^+^ cells. The NK super-cluster consisted of two NKT cell populations (*Cd3e*^+^) and two NK cell populations. Between the two NKT populations, differential gene expression analysis showed that cluster 16 expressed higher levels of *Ctla4,* while cluster 12 expressed more *Il2rb* and *Cxcr6*. When comparing the NK populations, cluster 15 expressed higher amounts of *Gzma* and *Ccl5,* while cluster 2 had a higher expression of *Hmgb1*. These patterns are indicative of activated (12 and 15) and unactivated (16 and 2) clusters within each pair (25, 26). Within the B cell super-cluster, naive and immature cells were differentiated by the expression of *Il7r*, which is only present on immature B cells (27). Early activated B cells decreased the expression of *Ighd* and *Sell*, and there was a population of activated B cells that expressed a high amount of *Ly6a*. Additionally, there was one plasma cell cluster identified by reduced expression of all B cell markers (28). The three myeloid subpopulations consisted of M1 macrophages (*Ccl6*^+^ and *Ccr2*^+^), M2 macrophages (*C1qa*^+^), and monocytes (*Cd52*^+^). Finally, there were singular clusters of plasmacytoid dendritic cells (pDC; *Siglech*^+^, *Itgax*^+^, and *Irf7*^+^), platelets (*Itga2b*^+^ and *Ppbp*^+^), and erythrocytes (*Hbb-bt*^+^ and *Hba-a1*^+^).

To examine the degree of heterogeneity between subclusters, the top six genes defining each subcluster (i.e., the top genes expressed uniquely within each cluster relative to the rest of the data set and commonly shared between uninfected and infected samples) were plotted on a heatmap with others from the same supercluster. T cells (Fig. 2B) and NK cells (Fig. 2C) displayed a large amount of heterogeneity, with the top genes in each cluster being exclusively or very highly expressed within that cluster. B cells (Fig. 2D) showed less heterogeneity between clusters, while myeloid cell (Fig. 2E) clusters 7 (monocyte) and 17 (M1 macrophage) showed similarity and cluster 5 (M2 macrophage) was distinct. Taken together, these data identify distinct cell populations in the circulating blood based on unique transcriptomic signatures before and after SINV infection of the CNS.

### Recruitment of circulating leukocytes into the CNS following SINV infection

**Fig 3:**
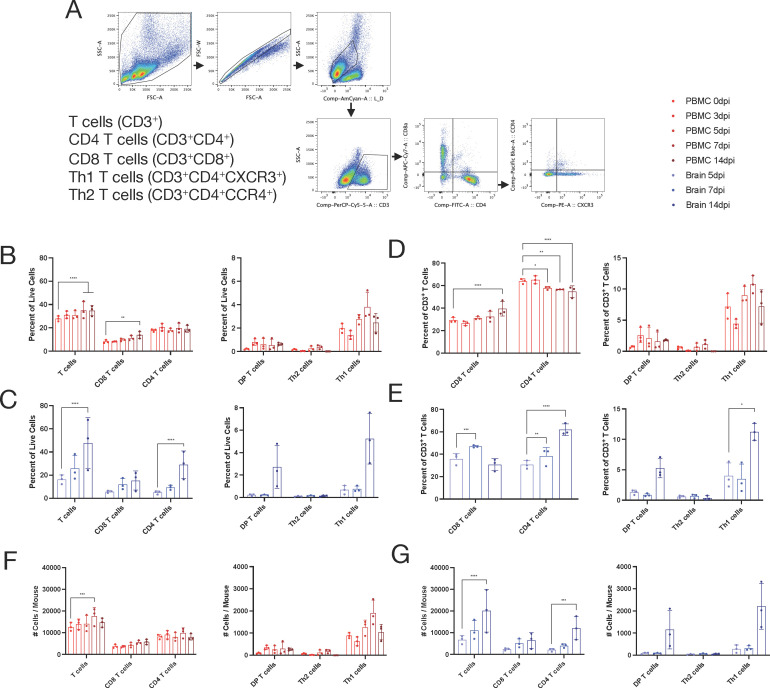
T cell population changes in the blood correlate with infiltration into the brain. (**A**) T cell gating scheme for analysis. (**B–G**) Identification by flow cytometry of T cells in the blood (red) or brain (blue) over the course of infection at 0, 3, 5, 7, and 14 dpi (blood) or 5, 7, and 14 dpi (brain). The subpopulations are presented as either a percentage of all live cells (**B and C**), a percentage of CD3^+^ T cells (**D and E**), or the total number of cells per mouse (**F and G**) with the mean ± SD from three independent experiments with the cells pooled from four to seven mice. No indicator, non-significant, *P < 0.05, **P < 0.01, ***P < 0.001, and ****P < 0.0001; Dunnett’s multiple comparisons test.

**Fig 4:**
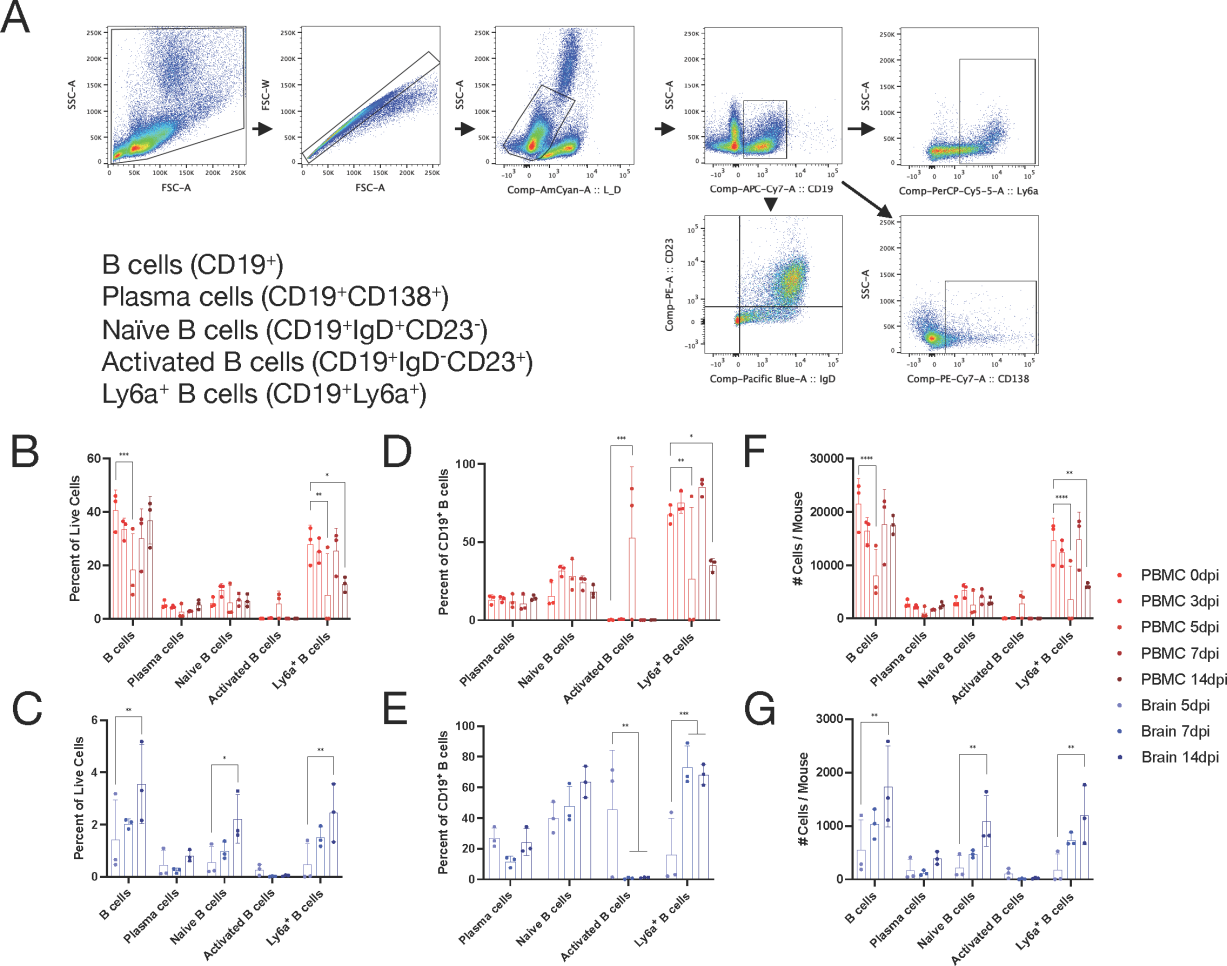
B cell population dynamics in the blood and brain. (**A**) B cell gating scheme for analysis. (**B–G**) Identification by flow cytometry of B cells in circulating blood (red) or brain (blue) over the course of infection at 0, 3, 5, 7, and 14 dpi (blood) or 5, 7, and 14 dpi (brain). The subpopulations are presented as either a percentage of all live cells (**B and C**), a percentage of CD19^+^ B cells (**D and E**), or the total number of cells per mouse (**F and G**) with the mean ± SD from three independent experiments with the cells pooled from four to seven mice. No indicator, non-significant, *P < 0.05, **P < 0.01, ***P < 0.001, and ****P < 0.0001; Dunnett’s multiple comparisons test.

Recovery from SINV infection of the CNS requires recruitment into the CNS of SINV-specific B cells producing antibodies and T cells producing IFNγ, which synergize to clear infectious virus and viral RNA (29–34). Therefore, we used flow cytometry to determine whether populations that appeared or disappeared from circulation were migrating to the CNS in response to infection. PBMCs were isolated from uninfected mice and infected mice at 3, 5, 7, and 14 dpi and analyzed by flow cytometry based on standard lymphocyte subsets as well as populations identified by scRNAseq (Fig. 3A and 4A). Infiltrating immune cells in the brain were analyzed at 5, 7, and 14 dpi as few lymphocytes were present in the CNS earlier during infection. T cells (Fig. 3; CD3^+^) were analyzed for CD4 T cells (CD3^+^CD4^+^), CD8 T cells (CD3^+^CD8^+^), Th1 T cells (CD3^+^CD4^+^CXCR3^+^), and Th2 T cells (CD3^+^CD4^+^CCR4^+^). Similarly, B cells (Fig. 4; CD19^+^) were analyzed for plasma cells (CD19^+^CD138^+^), naive B cells (CD19^+^IgD^+^CD23^-^), activated B cells (CD19^+^IgD^-^CD23^+^), and Ly6a^+^ B cells (CD19^+^Ly6a^+^).

T cells in the blood, as a proportion of live cells (Fig. 3B), showed a gradual increase over the course of infection (*P* < 0.001), which was driven by increased proportions of CD8^+^ T cells (*P* < 0.01) with little or no change in CD4^+^ T cells or CD4^+^CD8^+^ double-positive cells. Within the CD4^+^ T cell population, Th2 T cells (CCR4^+^) remained stable, and the proportion of Th1 T cells (CXCR3^+^) trended up in the early phase of infection (days 3–7), though it did not reach statistical significance. When looking at subpopulations as a share of total T cells (Fig. 3D), CD8^+^ T cells increased (*P* < 0.0001), while CD4^+^ T cells decreased (*P* < 0.0001).

As a share of live cells in the brain, total T cells doubled from 5 to 14 dpi (*P* < 0.0001), and this was mainly driven by an increase in CD4 T cells (*P* < 0.0001) (Fig. 3C). As a percentage of total T cells, CD8^+^ T cells increased through day 7 (*P* < 0.001), while CD4^+^ T cells continued to increase through the last sampling at 14 dpi (*P* < 0.0001) (Fig. 3E). Of the CD4^+^ T cells, Th1 cells were the primary contributors to the increase from 7 to 14 dpi (*P* < 0.05). Absolute numbers of each cell population followed similar trends to that observed for cells as a proportion of total cells in both blood (Fig. 3F) and brain (Fig. 3G). Overall, analysis of T cell representation in blood and CNS showed that after infection, T cells increased in blood with a greater increase in CD8 than CD4 T cells, while in the brain there was also a steady increase in T cells with more CD8 T cells early and CD4 T cells later. At both sites, there was a preferential enrichment of Th1 cells within CD4 T cells.

In the blood, B cells displayed unexpected dynamics (Fig. 4B, D, and F). Total B cells (CD19^+^) in circulation decreased after infection (*P* < 0.001) with a low at 5 dpi of about 50% of baseline and then increased to levels prior to infection by 14 dpi. Within the B cell population, this pattern was also observed in Ly6a^+^ B cells (*P* < 0.01) and coincided with an increase in activated B cells at the same time point (*P* < 0.001), but high variability is observed at the 5-dpi time point. As a share of the B cell population (Fig. 4D), plasma cells and naive B cells remained unchanged over the course of infection.

In the brain, all B cell subsets, as a proportion of live cells (Fig. 4C), increased from 5 to 14 dpi, and these cells included total B cells (*P* < 0.01), naive B cells (*P* < 0.05), and Ly6a^+^ B cells (*P* < 0.01), but not activated B cells or plasma cells. When examining the distribution of subsets within the B cell (CD19^+^) population (Fig. 4E), activated B cells (CD23^+^) were 40% of B cells at 5 dpi before essentially disappearing at 7 dpi and remaining low through 14 dpi (*P* < 0.01), albeit with large variability at 5 dpi, and Ly6a^+^ B cells increased from 20% to 70% (*P* < 0.001) during the same time.

Absolute numbers of each B cell population in PBMCs and brain (Fig. 4F and G) followed similar trends to cells as a proportion of total B cells. Overall, these results demonstrate observable changes to circulating immune cells following CNS infection with SINV, which may not have been predicted from the analysis of changes in CNS or CLN cell populations.

### Transcriptional signatures of PBMCs of SINV-infected mice

**Fig 5:**
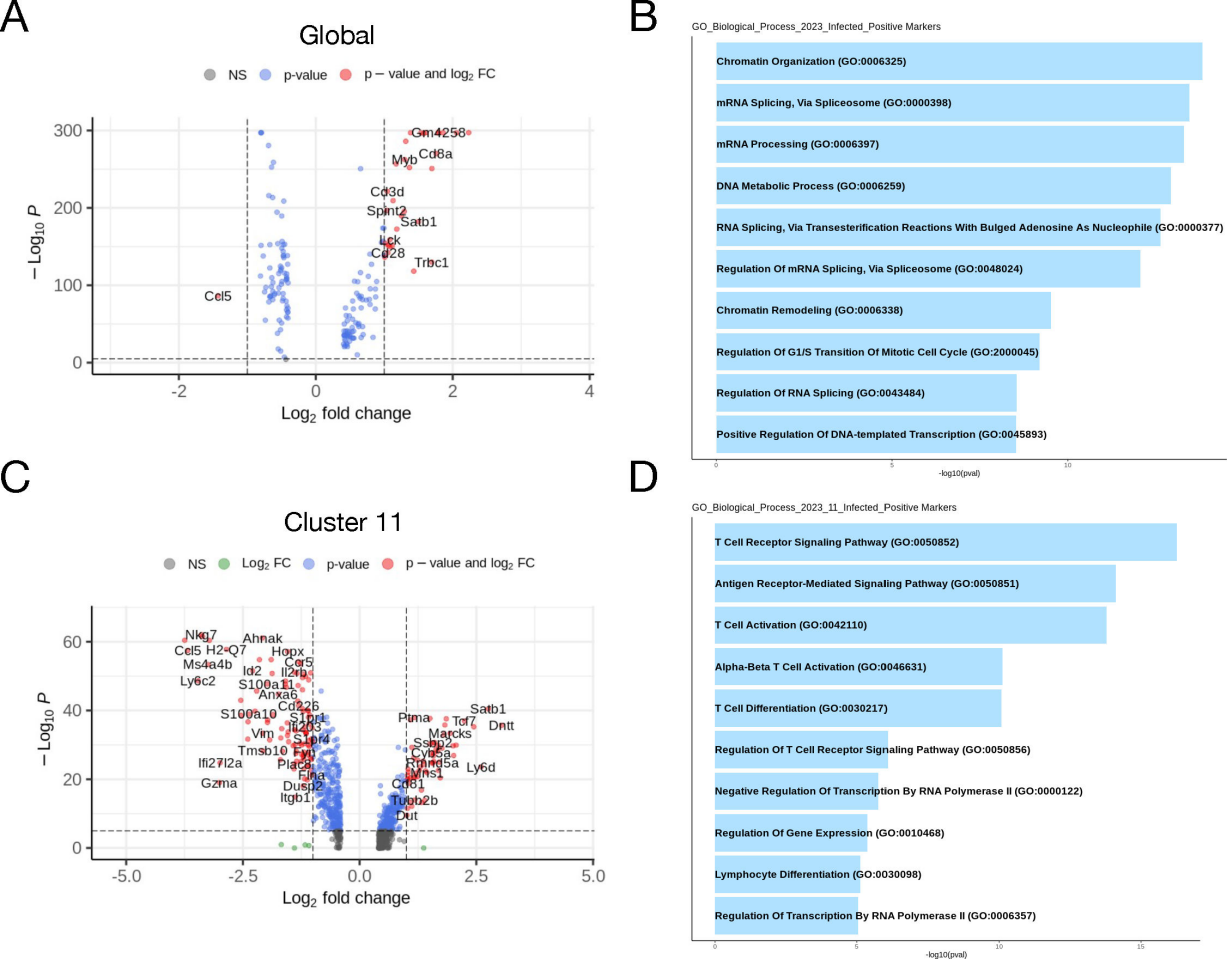
Transcriptomic signature of circulating immune cells during viral encephalitis. (**A and C**) Volcano plots representing differentially expressed genes between control and infected mice globally (**A**) or within cluster 11 (**C**). Results were calculated using the FindMarkers function in Seurat specifying the Wilcoxon rank-sum test with genes detected in 25% of cells and at least 1.5-log-fold change between groups. (**B and D**) Pathway visualization bar plot of differentially expressed genes using the GO biological process database.

We next assessed differential gene expression in the scRNAseq data between uninfected and infected mice to determine transcriptomic changes to PBMCs upon infection. This was done using the FindMarkers command in Seurat using the Wilcoxon rank-sum test with genes expressed in a minimum of 25% of cells and a log-fold change (LFC) minimum of 1.5. Globally (Fig. 5A), the only gene significantly upregulated in infected mice at 7 dpi compared to uninfected mice was *Ccl5*. Gene ontology (GO) analysis of upregulated genes in infected mice compared to uninfected mice (Fig. 5B) largely identified biological processes related to translation and ribosomal processing. Moreover, analysis of gene expression differences at the individual cluster level yielded substantive differences only in cluster 11 (Fig. 5C), the Ki67^+^ CD8 T cell population. Notable upregulated genes in PBMCs from infected mice include *Ccl5*, *Ccr5*, *Il2rb*, and *Gzma*. GO analysis of cluster 11 (Fig. 5D) identified upregulation of genes involved in chemotaxis/cell migration and T cell activation and differentiation.

**Fig 6:**
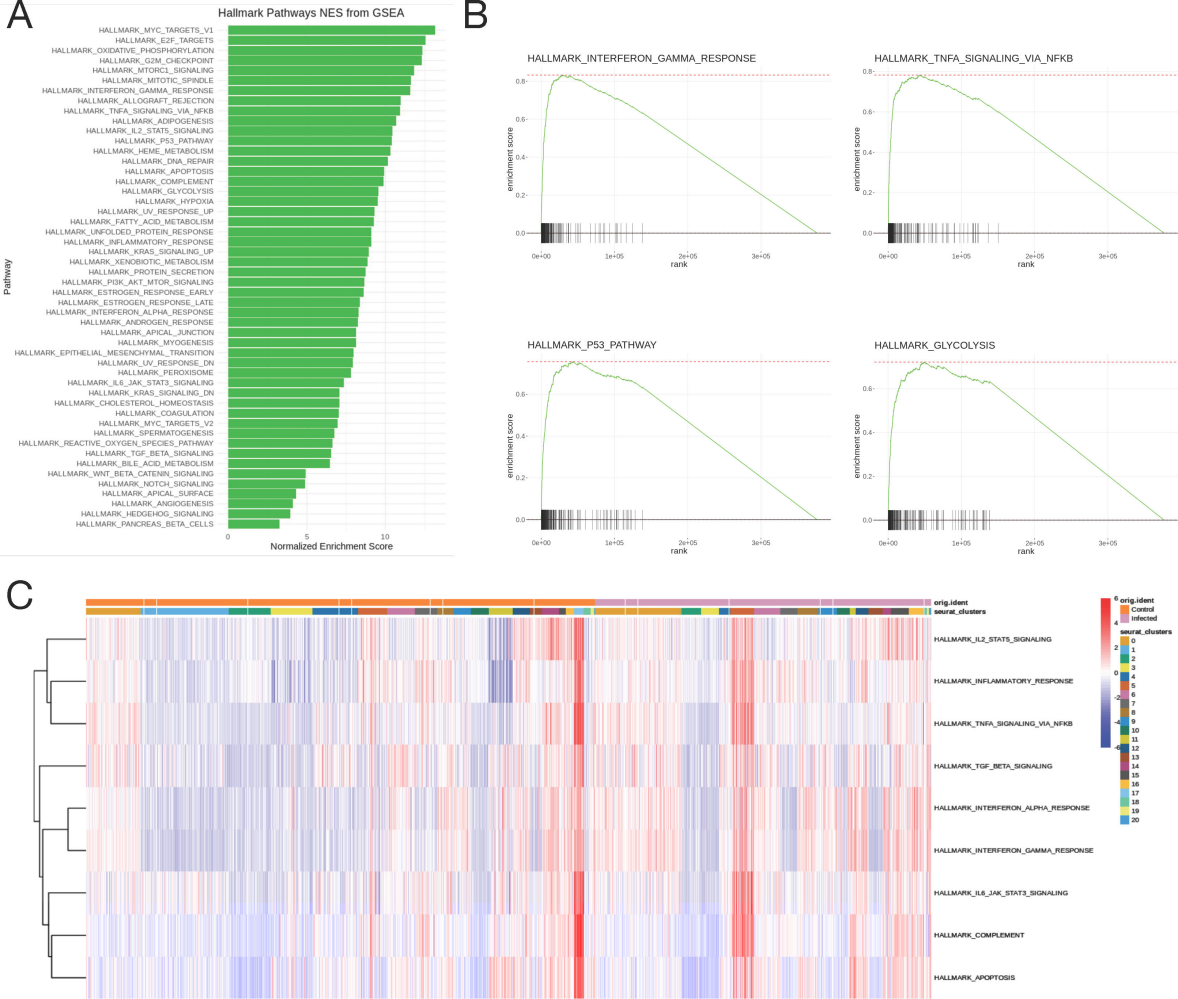
Gene set enrichment analysis. (**A**) Normalized enrichment scores from GSEA of the 50 hallmark gene sets from the MSigDB. (**B**) Enrichment plots for key immune pathways—IFNγ pathway (top left), TNFα pathway via NF-κB (top right), p53 pathway (bottom left), and glycolysis pathway (bottom right). The black lines represent the position of genes in each pathway in a ranked list of differentially expressed genes, and the area under the curve represents the total enrichment of response genes. (**C**) Heatmap showing enrichment scores of hallmark immune pathways in individual cells striated by cluster and infection status.

Global gene set enrichment analysis (GSEA) of differentially expressed genes in PBMCs from infected relative to control mice showed upregulation of gene sets related to inflammation/immune activation and mitosis (Fig. 6A). The normalized enrichment scores are shown for the 50 hallmark gene sets in the molecular signatures database (MSigDB), and every gene set is enriched in infected mice compared to uninfected mice. Enrichment plots (Fig. 6B) for key metabolic and inflammatory pathways show significant upregulation and enrichment in the infected mice, here displaying the IFNγ pathway (top left), TNFα pathway via NF-κB (top right), p53 pathway (bottom left), and glycolysis pathway (bottom right), an indicator of T cell activation (35). The area under the curve represents the enrichment of response genes. Additionally, indicative of leukocyte proliferation, the E2F target pathway was also enriched, along with the inflammatory response, IL-2/STAT5, and complement pathways (Fig. 6A). When looking at the enrichment of immune-related pathways at the cluster level (Fig. 6C), inflammatory pathways were most highly upregulated in clusters 5 and 17 (macrophages). These data identify inflammatory genes and pathways upregulated in PBMC populations following CNS infection.

### Peripheral cytokine expression during CNS-restricted alphavirus encephalomyelitis

Finally, due to the identification of a dynamic response in the blood during viral infection of the CNS, we asked whether any upregulated gene products could be detected in the serum. SINV infection in humans is generally diagnosed by serological techniques, such as enzyme-linked immunosorbent assay (ELISA) for virus-specific IgM or IgG; however, seroconversion does not always coincide with the onset of symptoms (36, 37), which complicates the diagnostic process. Similarly, RT-PCR-based methods are handicapped by a short and low viremic period in humans (36, 38).

**Fig 7:**
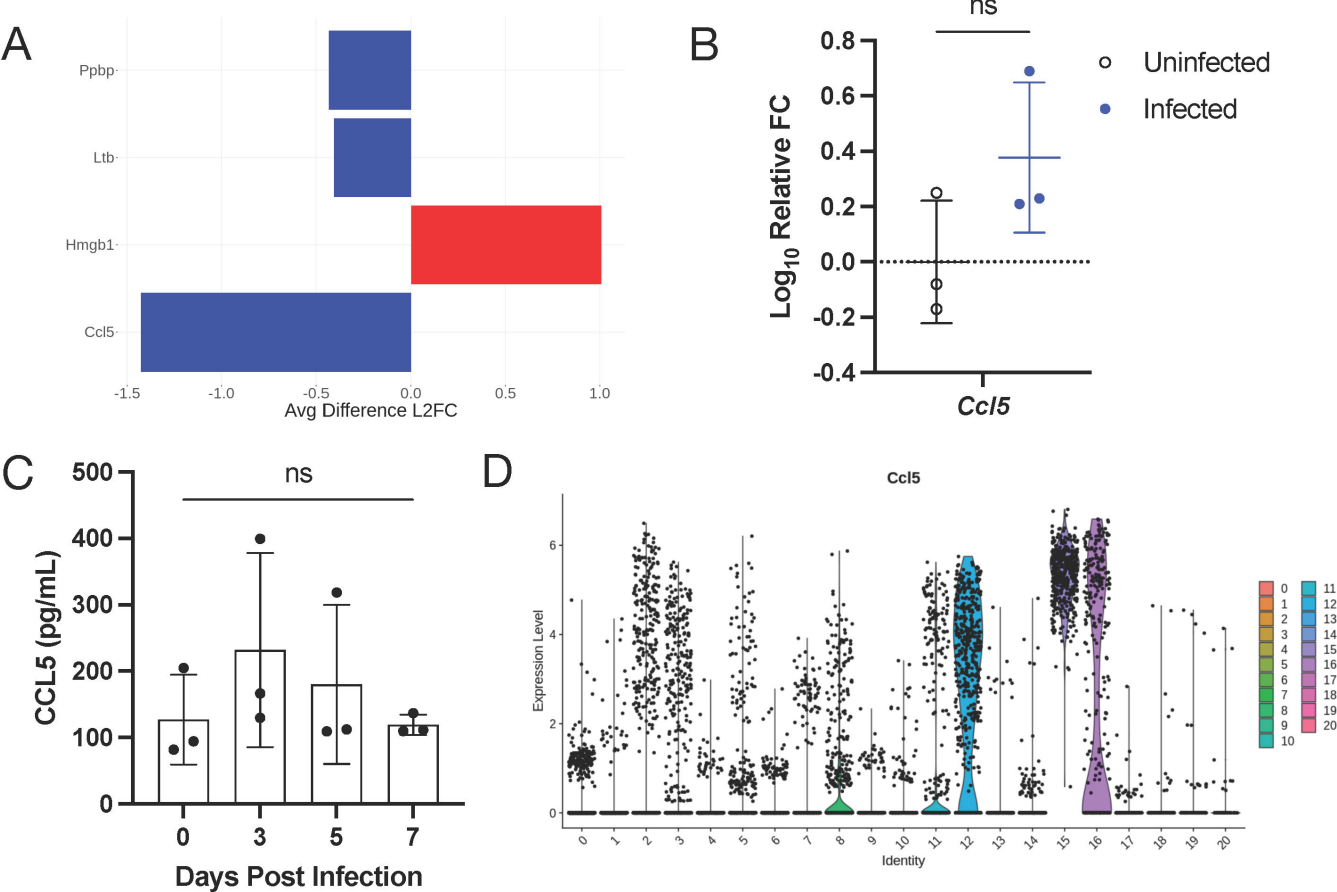
Serum cytokines of viral encephalitis. (**A**) Differentially expressed cytokines in scSeq data with at least 1.5 LFC relative to the entire data set and expressed in at least 25% of cells. Data are presented as differences in LFC between control and infected, where negative numbers indicate higher expression in the infected mice. (**B**) qRT-PCR for *Ccl5* expression in all PBMCs. Data are presented as the mean ± SD from two independent experiments with the cells pooled from four to seven mice. (**C**) Serum levels of CCL5 detected by ELISA. (**D**) Expression of *Ccl5* striated by individual clusters. ns, non-significant; unpaired *t*-test (qRT-PCR) and Tukey’s multiple comparisons test (ELISA).

We first analyzed the data globally for differential expression of cytokine and chemokine mRNAs. Four genes were differentially expressed above the 1.5 LFC threshold: *Ccl5* (RANTES), *Ppbp* (CXCL7), and *Ltb* (lymphotoxin B) were upregulated and *Hmbg1* was downregulated (Fig. 7A), with *Ccl5* being the most highly upregulated cytokine in PBMCs at 7 dpi. To confirm the upregulation of *Ccl5* mRNA, total RNA from PBMCs was analyzed by RT-qPCR for relative change in expression after infection. *Ccl5* was transcriptionally upregulated following SINV infection with a roughly 0.40-fold change (Fig. 7B). Protein levels of CCL5 in the serum were measured by enzyme-linked immunosorbent assay at 0, 3, 5, and 7 dpi. CCL5 was increased at 3 dpi before gradually returning to baseline levels by 7 dpi (Fig. 7C). While both RNA and protein levels of CCL5 showed an upward trend from baseline, neither reached statistical significance. *Ccl5* was expressed mainly by NK and NKT cells (clusters 12, 15, and 16; Fig. 7D). CCL5 is an inflammatory chemokine that recruits leukocytes to the site of infection and can also be a potent leukocyte activator (39). These data suggest that CCL5 is an important chemokine in the periphery during response to SINV encephalomyelitis.

## DISCUSSION

The immune response to alphavirus encephalomyelitis is initiated in the draining CLNs before the recruitment of immune cells into the brain and spinal cord (17, 40); however, whether infection induces detectable transcriptomic or immunologic changes to circulating immune cells remains understudied. The current studies combined data from scRNAseq and flow cytometry to show that viral infection of the CNS causes changes to the composition of cells in circulation indicative of proliferation, immune activation, and inflammation. Changes to PBMC composition identified by scRNAseq clustering showed an increase in naive B cells and a decrease in immature T cells in mice at 7 days after intracerebral infection with SINV compared to uninfected mice. GO analysis showed upregulation of genes related to translation in the PBMCs of infected mice compared to uninfected mice. At the cluster level, only Ki67^+^ CD8 T cells showed significant changes to gene regulation and turned-on genes involved in cell migration/chemotaxis and T cell activation with enrichment of inflammatory, cell cycle, and metabolic pathways. Longitudinal analysis of PBMCs by flow cytometry confirmed these findings and identified increases in total T cells that were driven by CD8 T cell increases with little change in CD4 T cells. Within the CD4 population, Th1 cells were most highly represented. In the brain, however, all populations of T cells steadily increased after infection, except Th2 cells. Most B cells in circulation initially decreased after infection before returning to baseline levels by 14 dpi. One notable exception was activated B cells, which increased transiently at 5 dpi, but high variability prevents firm conclusions. This is contrasted by a steady increase of all B cell populations in the brain from 5 to 14 dpi. Transcriptional changes included a slight upregulation of *Ccl5* from baseline, which was also modestly increased in serum. Therefore, distinct dynamic immune cell changes can be identified in the blood as well as the CNS during viral encephalomyelitis.

Viral clearance and disease onset correlate with infiltration of T cells into the brain (41), which are generally not present in the disease-free CNS (42). Therefore, there are likely secreted factors originating from antigen-experienced cells that mobilize mononuclear cells in the periphery, and chemotaxis plays an important role in cellular translocation. For example, SINV infection induces the expression of chemokine CCL1, CCL2, CCL5, CXCL9, CXCL10, and CXCL13 mRNAs in both the brain and spinal cord (20, 43), which recruit innate and adaptive immune cells into the CNS. This is consistent with the upregulation of chemotaxis-related pathways in a subset of PBMCs (Fig. 5D). Additionally, lymph node cells produce mononuclear chemotactic factors following SINV infection, which attract peritoneal exudate cells (44), and adoptive immunization with SINV-sensitized lymph node cells reconstitutes the CNS inflammatory response in immunosuppressed mice (40). Taken together, this implies that cells in circulation respond to chemotactic factors to facilitate their migration to the CNS and possibly CLNs in response to viral infection.

This modulation of PBMCs following infection can be detected at the cellular level. T cell kinetics follow an expected pattern for acute viral infection, where Th1 cells continually increased through day 14 after infection (Fig. 3) consistent with previous reports that both CD4 and CD8 T cells are recruited to the brain following SINV infection and continually increase through 10 dpi (45, 46). During SINV infection, recruited T cells are canonically Th1 due to their production of IFNγ, but they also produce the inflammatory cytokines TNFα, IL-2, and IL-17a (47). As such, the expansion of Th1 cells in the blood coincides with their appearance in the CNS (Fig. 3). The T cell response to SINV infection is paradoxical: T cell sourced IFNγ promotes viral clearance, but the onset of neurologic disease and inflammation is concurrent with the arrival of T cells into the CNS (6). Although clearance of SINV from most neuronal populations is dependent on the humoral response, IFNγ is sufficient to clear the virus from the spinal cord and synergizes with antibodies to clear the virus from the brain (32, 48). However, T cells also contribute to mortality, as mice deficient in mature T cells survive infection with the virulent neuroadapted strain of SINV, whereas immunocompetent mice do not (49). This effect is mainly driven by pathogenic Th17 cells that are dampened by IL-10 expression (41). In other models of viral encephalitis such as infection with rabies and West Nile virus, peripheral T cell dynamics are similar for total T cells (50, 51), suggesting that the neuroinflammatory process involves the recruitment of activated T cells from the circulation.

On the other hand, B cells showed an unexpected decrease in the blood at 5 dpi when they became detectable in the brain followed by a rebound in PBMCs and continued to increase in the CNS through 14 dpi (Fig. 4). SINV-specific antibody-secreting cells (ASCs) are specifically retained in the CNS, as their proportion of total ASCs rises from 15% at day 14 to 90% at 4–6 months (16). Retention is correlated with sustained expression of IL-10 and IL-21 mRNAs, cytokines important for B cell proliferation and differentiation (43), which may be facilitated by the recruitment of Treg cells as a source of IL-10. A lesser decrease in B cells in PBMCs following SINV infection has previously been reported in BALB/c mice (52), followed by an increase in spleen SINV-specific ASCs up to 7 dpi (53), followed by a decline indicative of their egress into circulation and consistent with our observation that B cells start to rebound after 5 dpi (Fig. 3). Similar B cell kinetics have been observed during infection with the neurotropic JHM strain of mouse hepatitis virus, a coronavirus, but PBMCs were not examined (54, 55). Even less is known about B cell kinetics in the blood for other viral encephalitis models such as infection with Semliki Forest, Venezuelan equine encephalitis, and Theiler’s murine encephalomyelitis viruses. West Nile virus infection in a rhesus macaque model causes an initial decrease in proliferating B cells (CD20^+^Ki67^+^) by roughly 5%–10% in the CD3^-^ pool of circulating cells before increasing after 4 dpi (56). Interestingly, the same kinetics are observed with B cells following infection with *Plasmodium chabaudi*, an apicomplexan parasite that infects hepatocytes and erythrocytes (57). Collectively, the initial reduction in circulating B cells could be due to their migration to peripheral lymphoid tissue, such as the CLN or spleen to participate in the antibody response (29), but further studies examining PBMCs in mice are needed. During acute viral infection of the CNS, viral antigens trafficked to the CLNs’ activation of naive B cells that then become ASCs or memory B cells (58). The time between initial infection and activation is canonically 7–10 days for extrafollicular B cells and 10–14 days for germinal center B cells (59). Thus, an initial reduction in B cells from the circulating pool before rebound at 7 dpi following SINV infection (Fig. 3) is likely indicative of their trafficking to and activation in the CLNs, and their re-entry into circulation post-activation coincides with the appearance of anti-SINV IgM and IgG in serum and B cells in the CNS (20, 32, 34).

Finally, the presence of large proportions of Ly6a^+^ B cells in both sequencing and flow cytometric data (Fig. 2 and 4) was surprising despite high variability in the blood at 5 dpi. Ly6a, also known as Sca1, is a glycosyl phosphatidylinositol-anchored cell surface protein that is generally used as a marker for hematopoietic stem cells. It is a receptor for an unidentified ligand, and the majority of phenotypes in Ly6a knock-out mice appear to be associated with processes involving tissue-resident stem/progenitor cells (60). Additionally, overexpression of Ly6a on T cells causes self-aggregation and adherence to B and T cells *in vitro*, suggesting that lymphocytes express the ligand for Ly6a (61). Ly6a is also expressed on brain microvascular endothelial cells and is implicated in adeno-associated virus (AAV) crossing of the blood-brain barrier (BBB) (62). Little is known about the function of Ly6a in B cells. Studies comparing Ly6a^+^ and Ly6a^-^ B cells reveal a potential role in differentiation, as the majority of ASCs were Ly6a^+^ (63), and in Ly6a-deficient mice, there is an increased B cell expression of the immunoglobulin λ light chain (64). Further studies are needed to determine the role of Ly6a^+^ B cells in response to CNS infection and whether the observed pattern is specific to CNS infection or is a general B cell response. However, due to the association of Ly6a expression by brain endothelial cells and AAV passage across the BBB, we speculate that Ly6a expression is involved in B cell transmigration into the CNS.

Modulation of immune cell representation in PBMCs during VE can also be detected at the transcriptomic level. At the 7 dpi time point, naive B cells (cluster 0) were present only in infected mice and immature T cells (cluster 1) in uninfected mice (Fig. 1D), which is consistent with flow cytometry observations showing expansion of naive B cells and mature T cells after infection (Fig. 3 and 4). In addition to the expansion of these populations, differential gene expression analysis also shows immune activation of circulating leukocytes. For example, Ki67^+^ CD8 T cells (cluster 11) upregulate the expression of genes related to cell migration/chemotaxis, cellular differentiation, and T cell activation, which include genes such as *Ccl5*, *Ccr5*, *Gzma*, and *Il2rb* (Fig. 4). *Ccl5* and *Ccr5* expression is consistent with what is known about recovery from SINV infection. CD8 T cells are the first virus-specific lymphocytes to enter the brain (16) and can do so by CCL5-mediated chemotaxis, which is upregulated in the CNS following SINV infection (20, 65). Additionally, upregulation of the IL-2 receptor, *Il2rb*, allows for T cell activation by IL-2, which is also present in the CNS of SINV-infected mice (47). Finally, despite higher expression of *Gzma* in infected mice, T cell-mediated recovery from SINV tends to facilitate viral clearance by non-cytolytic mechanisms such as the production of IFNγ (48, 66). This is reflected in the pathway analysis, where the IFNγ response is the fourth highest enriched pathway, in addition to the TNF, NF-κB, and inflammatory pathways (Fig. 5), which are also consistent with recovery from SINV infection. These pathways are similar to those identified in humans infected with chikungunya virus (67) and transcriptional signatures in infected C57BL/6J mice (68) and in PBMCs of patients with influenza virus-associated encephalopathy (69). Collectively, observable immune activation in circulating immune cells is associated with the inflammatory response to SINV infection of the CNS, again suggesting that activated lymphocytes re-enter the circulation prior to their trafficking to the CNS. It is likely that cytokines and chemokines expressed and secreted into the blood early after pathogen recognition mobilize a faster immune response.

*Ccl5* was the most highly upregulated cytokine mRNA in PBMCs of infected mice at 7 dpi compared to uninfected controls, with CCL5 protein levels highest in serum at 3 dpi (Fig. 7), albeit only modestly (20). Consistent with early production, *Ccl5* was mainly expressed by NK cells (25). Although IFNγ^+^ NK cells infiltrate into the CNS during SINV infection, no clear role for them has been defined in the recovery process (46, 70, 71). CCL5, also known as RANTES, facilitates the trafficking of T cells, monocytes, dendritic cells, and NK cells, all of which have been associated with the immune response to SINV (6, 33, 65). Therefore, it is likely that NK-sourced CCL5 augments the trafficking of leukocytes to the CNS. CCL5 expression is characteristic of many infections of the CNS, including West Nile virus, Venezuelan equine encephalitis virus, tick-borne encephalitis virus, and the parasite, *Toxoplasma gondii* (72–75); however, specific expression by PBMCs has not been explored in this context.

In summary, immune activation can be observed in PBMCs following a CNS-restricted viral infection. PBMCs upregulate inflammatory, proliferative, and chemotactic genes while in circulation and, presumably, before their trafficking to the CLNs or CNS. These observations identify changes in PBMCs during viral encephalitis, such as the production of CCL5 and a transient B cell lymphopenia, which will inform studies of the pathogenesis of infection in humans and highlight the importance of examining immune responses in multiple compartments in addition to the tissue of interest.

## MATERIALS AND METHODS

### Sindbis virus infection of mice

C57BL/6J (WT) mice were obtained from Jackson Laboratories and bred in-house. The SINV TE strain (12) was grown on BHK-21 cells and quantified by plaque formation. For infection, 4–6-week-old mice of both sexes were briefly anesthetized with isoflurane and intracranially inoculated in the left cerebral hemisphere with 10^3^ PFU of virus diluted in 20 µL of phosphate-buffered saline (PBS).

### Mononuclear cell isolation

For PBMCs, heparinized blood was collected via cardiocentesis from three uninfected control mice and three infected mice at 7 dpi. PBMCs were isolated from the pooled blood samples by whole blood centrifugation on a Ficoll-Paque PLUS (Cytiva) density gradient. Mononuclear cells were collected at the interface, erythrocytes were lysed with ACK lysing buffer (Quality Biological), and cells were washed several times with RPMI (Gibco) containing 1% fetal bovine serum (FBS) before freezing in a 10% DMSO and 90% FBS solution.

For CNS tissue, single-cell suspensions were made from freshly isolated brains by homogenizing three times in RPMI containing 1% FBS, 1 mg/mL collagenase IV (Worthington Biochemical Corporation), and 0.1 mg/mL DNase I (Stem Cell Technologies) using the GentleMACS system (Miltenyi), with 15 min 37°C incubation and gentle agitation between each round of homogenization. Homogenates were filtered through 70 µm filters and washed with RPMI containing 1% FBS. Myelin debris and red blood cells were removed by centrifugation on a 30%/70% percoll gradient for 30 min at 4°C. Mononuclear cells were collected at the interface, erythrocytes were lysed with ACK lysing buffer (Quality Biological) and washed several times with RPMI containing 1% FBS before freezing in a 10% DMSO and 90% FBS solution.

### Library construction and sequencing

Freshly isolated PBMCs from each group were loaded with barcoded gel beads onto a Chromium Chip A to produce single-cell gel bead-in-emulsions. Cells were lysed following the 10× Genomics protocol, and transcripts were reverse transcribed. Single-cell capture and library creation were performed using the Chromium Single Cell 3′ Reagent kit v3 according to 10× Genomics specifications. Full-length cDNA with cell-barcode identifiers were PCR amplified, and libraries were normalized to 4 nM before being sequenced on the Illumina Novaseq. All library preparation and sequencing were done with help from the Johns Hopkins Single Cell and Transcriptomics Core.

### scSeq data demultiplexing and clustering

10× Genomics Cell Ranger version 4.0 (76) was used to perform demultiplexing, process barcodes, UMIs, and align to the mouse genome mm10 (GCF_000001635.20) via STAR (77). Using the Seurat R package (78), unwanted cells were filtered out based on mitochondrial gene representation and variances in unique gene counts. Doublets were predicted and removed using DoubletFinder (79). A total of 8,608 and 5,696 cells were left, respectively, for the control and SINV-infected mouse cells. The data sets were normalized and integrated using Seurat and followed with PCA and UMAP dimension reduction. The data were clustered using the FindClusters function in Seurat at a resolution determined by Clustree (21). Cell clusters were annotated manually by expression levels and distributions of population-specific immune cell markers after automated annotation by UNCURL (22) based on the top genes for each cluster identified through the FindConservedMarkers function in Seurat. Differential gene expression was done using the FindMarkers function in Seurat, and GSEA was done using the Escape package (80).

### Flow cytometry and intracellular staining

After mononuclear cell isolation, cells were thawed, washed, counted, and 10^6^ cells were plated to be stained with the LIVE/DEAD Fixable Aqua Stain (Invitrogen) in PE buffer (1× PBS, 2 mM EDTA), blocked with rat anti-mouse CD16/CD32 (BD Pharmingen, clone 2.4G2) diluted in PE buffer, and surface stained with an antibody cocktail in FACS buffer (1× PBS, 2 mM EDTA, and 0.5% BSA) on ice for 30 min. The following antibodies from BioLegend, BD Pharmingen, or Invitrogen were used for surface staining: CD4 (clone RM4-5), CD183 (CXCR3; clone CXCR3-173), CD3 (clone 17A2), CD8a (clone 53-6.7), CD194 (CCR4; clone 2G12), CD23 (clone B3B4), Ly6A/E (clone D7), CD138 (clone 281-2), CD19 (clone 6D5), and IgD (clone 11-26c.2a).

Following staining, cells were washed and resuspended in FACS buffer, and data were acquired on 100,000 cells per sample using a BD FACS Canto II flow cytometer with FACS Diva software (version 6.0) and analyzed using FlowJo (version 10.8) with bad events removed by the FlowAI plugin and gating based on fluorescence minus one. Cell types were defined as follows: B cells (CD19^+^), plasma cells (CD19^+^CD138^+^), naive B cells (CD19^+^CD23^-^IgD^+^), activated B cells (CD19^+^CD23^+^IgD^-^), Ly6a^+^ B cells (CD19^+^Ly6a^+^), T cells (CD3^+^), CD4 T cells (CD3^+^CD4^+^), CD8 T cells (CD3^+^CD8^+^), Th1 T cells (CD3^+^CD4^+^CXCR3^+^), and Th2 T cells (CD3^+^CD4^+^CCR4^+^).

### Real-time quantitative reverse transcription PCR

After mononuclear cell isolation, RNA was isolated from PBMCs using the RNeasy Plus Mini kit (Qiagen) and quantified with a nanodrop spectrophotometer. cDNA was synthesized using the SuperScript III First-Strand Synthesis System (Invitrogen) with normalized RNA concentrations across samples and oligo dT primers according to the manufacturer’s instructions. After diluting the cDNA, quantitative real-time PCR was performed using PrimeTime gene expression assays (Integrated DNA Technologies) and EagleTaq Universal Master Mix (Roche). *Gapdh* mRNA levels were determined using the TaqMan Rodent GAPDH Control Reagents (Applied Biosystems). All reactions were run on an Applied Biosystems 7500 real-time PCR machine. The target gene Ct value was normalized to the Ct value of *Gapdh*. This normalized value was used to calculate the gene expression level relative to the average of the uninfected control value.

For SINV RNA quantification, RNA was isolated from PBMCs as stated above and from previously frozen brains that were homogenized in Lysing Matrix D tubes with 1 mL Qiazol at 6.0 M/s for 40 s in a FastPrep-24 homogenizer (MP Biomedicals), followed by RNA extraction using the RNeasy Plus Mini kit (Qiagen). cDNA was synthesized as stated above, and RT-qPCR was performed using EagleTaq Universal Master Mix (Roche) and the following primers and probe specific for SINV *nsP2*: primer nsP2 3373F (5′-CCG CAA GTA TGG GTA CGA TCA-3′), primer nsP2 3454R (5′-GTG CCC TTC CCA GCT AGC T-3′), and TaqMan probe nsP2 3317 [5′–6-carboxyfluorescein (6-FAM)–CCA TTG CCG CCG AAC TCT CCC–6-carboxytetramethylrhodamine (6-TAMRA)–3′]. *Gapdh* mRNA levels were determined using the TaqMan Rodent GAPDH Control Reagents (Applied Biosystems). All reactions were run on an Applied Biosystems 7500 real-time PCR machine. RNA copy numbers were quantified using a standard curve consisting of 10-fold serial dilutions ranging from 3  ×  10^7^ to 300 copies of the pCRII-TOPO plasmid containing the SINV nsP2 region and normalized to those for *Gapdh*.

### Enzyme-linked immunosorbent assay

Blood was collected from individual mice via cardiocentesis, and serum was separated in Microtainer SST tubes (Becton Dickinson) before storage at −80°C. Commercial ELISA kits were used to measure RANTES/CCL5 (Invitrogen) levels following the manufacturer’s instructions. Samples were tested at 1:4 dilution in duplicate.

### Statistics

Statistical analyses were performed using GraphPad Prism 10. Flow cytometry time course assays were analyzed by two-way analysis of variance (ANOVA) with Dunnett’s multiple comparisons post-test comparing to either 0 dpi (blood) or 5 dpi (brain). The ELISA time course assay was analyzed by one-way ANOVA with Tukey’s multiple comparisons post-test. qRT-PCR was analyzed by unpaired Student’s *t* test. A *P*-value of <0.05 was considered significant for all tests.

## Data Availability

The scRNAseq data for this study are available in SRA under BioProject number PRJNA1049823. The analysis packages used were all publicly sourced. Processed counts, code, and metadata are available on GitHub at https://github.com/b-hnguyen/sinv-pbmc-scseq
